# Bibliographical Notices

**Published:** 1847-11

**Authors:** 


### BIBLIOGRAPHICAL NOTICES.

*On Diseases of the Skin.* By ERASMUS WILSON, F. R. S., Consulting Surgeon to the St. Pancras Infirmary; Lecturer on Anatomy and Physiology in the Middlesex Hospital School of Medicine, etc. etc. *Second American from the second London edition.* 8vo. pp. 439. Lea & Blanchard. Philadelphia: 1847.

Although we have many works on Dermatology, we can hardly be said to have yet arrived at any great degree of accuracy in our diagnosis of the various affections, and, as a consequence, their treatment remains for the most part empirical and often unsatisfactory. The greatest confusion exists in the classifications of authors. Affections are described as distinct diseases, which often co-exist, or become blended, so as to bid defiance to all attempts at satisfactory diagnosis. With some, the *topographical system*, which divides the diseases according as they affect the head or the body generally, has been prefixed; but more generally the system of Plenck, modified and improved by Willan, was for a long time adopted. It consists of *six orders*, characterized by the appearance of the eruption, which, instead of constituting the disease, is in fact merely one of its products. To this succeeded the so-called natural system of Alibert, which embraced all cutaneous diseases in one group, under the name of *Dermatoses.* This constituted the trunk of his "Arbre des Dermatoses," the sub-groups being its branches. Although its gifted author assumed this to be the *Natural system,* in opposition to that of Willan, which has been designated the "*Artificial system,*" it was really not any more simple, and hardly more correct in reference to the phenomena of the varions affections. The great fault of Willan was in endeavouring to carry out the principles of division and subdivision pursued in Botanical and Zoological classifications; as if any analogy could exist between morbid conditions of a part of the human body and the natural or organic structure of plants and animals. In the attempt, affections the most dissimilar, as purpura and scarlatina, scabies and variola, were arranged together; whilst some of the most analogous, as variola and varicella, were separated. Our author adopts a new classification of his own, which he likewise proposes to call a "*Natural system,*" though it differs greatly from that of Alibert, and is, we think, with much greater reason entitled to that character. According to Mr. Wilson's arrangement, Diseases of the Skin are thrown into four groups, viz.: 1, Diseases of the Derma; 2, Diseases of the Sudoriparous Glands; 3, Diseases of the Sebiparous Glands; 4, Diseases of the Hairs and Hair Follicles.

What is called the skin, consists, as we know, of various structures, having their appropriate functions; and this classification has reference to the particular organs of the skin which are especially implicated. Disease in these several organs again is various in its character; thus we have inflammation, which may be either specific, non-specific, asthenic, sthenic, &c. &c.; augmented secretion, diminished secretion, retention of secretion, &c. &c. Hence, the classification is based on the distinct anatomical characters of the organs involved; which certainly is the most natural ground of separation. The particular character of the diseased action which occurs in these different structures, forms again a very natural and proper ground for subdivision.

If this classification were generally adopted, and we think it will be, it would do much to remove the confusion which exists in the diagnosis and pathology, as well as the therapeutic management of cutaneous affections.

The first chapter of the work contains an admirable account of the "Anatomy and Physiology of the Skin," embracing all the modern microscopical discoveries of the intimate structure of the derma, epiderma, and the included organs. This constitutes an appropriate, indeed, indispensable, introduction to the study of the diseases of which the work treats.

The concluding chapter contains a "history and description of the *itch animalcule*, or acarus scabei," and a "history and description of the *steatozoon folliculorum*," embracing some original and very curious observations.

We have read this edition of Wilson on Diseases of the Skin with unusual interest, aad cannot hesitate to recommend it as the most complete and satisfactory work on the subject now extant. The value of the volume is much increased by the excellent illustrations it contains; these consist of eight highly finished plates, representing groups of diseases, and comprising in all sixty-one different affections.

---

*A Treatise on the Structure, Diseases and Injuries of the Blood-vessels, with statistical deductions; being the Essay to which the Jacksonian prize for the year* 1847 *was awarded by the Royal College of Surgeons of England, with numerous additions.* By EDWARD S. CRISP, M. R. C. S., Member of the Council of the Pathological and Medical Societies of London, &c. &c. 8vo. pp. 354. London: 1847. *With five Lithographic Plates.*

The structure, diseases and injuries of the blood-vessels is an interesting topic to the physician and surgeon, but to the latter more especially: and we are, therefore, not astonished that it should be made the subject of a prize essay by the Royal College of Surgeons of England. From Mr. Crisp's account, fifty-two deaths occurred in the British metropolis in 1846 from aneurism; and according to the Report for 1840 of the Registrar-General, one hundred and forty-seven persons died from aneurismal diseases in England and Wales for that year,—a small proportion, certainly, for so large a population, when we compare it with the number of aneurisms in one year in the metropolis.

Two works on the diseases of the blood-vessels have been chiefly known amongst us:—one by Mr. Hodgson, of Birmingham, England, which was published upwards of thirty years ago; and that of our countryman Professor Nathan R. Smith, of Baltimore, on the Surgical Anatomy of the Arteries, the first edition of which was published about fifteen years ago. Both of these are valuable productions—doing great credit to their authors; but still there was sufficient room for the Essay before us; and the fact that it received the prize awarded by the Royal College of Surgeons of England for the year 1847, is sufficient evidence of its being possessed of real merit. Mr. Crisp states, that he has, for many years, paid much attention to this class of diseases, and that his residence in the neighbourhood of two large hospitals has afforded him many opportunities for witnessing operations, and watching the progress of these affections.

The descriptions of the different diseases are those generally received, although they are in some cases too brief. The pathological deductions are usually accurate; whilst the treatment is judicious. The author has taken considerable pains, too, in obtaining statistical details;—for instance, one single table of 551 spontaneous aneurisms, (so called) selected indiscriminately from the British Medical and Surgical Journals from the year 1785 to the present time, occupies a space of nearly forty closely printed pages. The table contains the authorities for each case, the age, sex, artery affected, habits, occupation, supposed cause, &c., operation, termination, cause of death, &c., and the name of the practitioner under whose care it fell.

To the work are prefixed five lithographic plates, the first representing an "arterial tree;" indicating by figures the order of frequency of the different situations of aneurisms; the others elucidating the structure of arteries, and certain lesions of the vessels. They are not signally good specimens of art.

*Criticisms and Controversies relating to the Nervous and Muscular Systems.* By Bennet Dowler, M. D., of New Orleans. *Reprinted from the New Orleans Medical and Surgical Journal, Sept.,* 1847.

This is a *brochure* of 69 pages, and racy enough for any palate—rather too spicy, indeed, for our taste.

On different occasions we have noticed Dr. Dowler's labours in terms of just praise. His experiments on the *cadaver*, in relation to caloricity and the influence of the nerves in causing muscular contraction, we regard as ingenious, and their results strange and inexplicable, according to present received physiological laws. These experiments, Dr. D. maintains, completely upset Dr. Marshall Hall's theory of reflex nervous action. This has drawn down upon his head the thunder of some of the admirers of the reflex doctrine, and the present publication is Dr. Dowler's rejoinder. It will not be denied, even by those who may not agree with the author in his reasonings, that his publication displays much learning and ability, but a sad want of the calmness of true philosophy. We admit that he has had great provocation. His experiments have been lightly spoken of, and his deductions have been put aside with a flippancy calculated to ruffle one unused to such encounters; but in losing his temper he sacrifices an important element in the controversy. We agree with him in thinking that he has not been fairly dealt with by his opponents. His experiments have been numerous, laborious, and seemingly accurately observed, and until others in an equally pains-taking manner have gone over the same ground, with different results, we are bound to regard them as true. No one, as far as we know, has denied that they are true. That being the case, his opponents are bound to show either that their theory is consistent with the facts, or that the facts stated are inapplicable to the case—in other words, that Dr. Dowler's deductions from his own premises are wrong. Thus far, ridicule rather than argument has been the weapon mostly employed against him.

